# Penetration of duodenal wall by proximal end of biliary straight plastic stent in a patient with ampullary carcinoma

**DOI:** 10.1002/deo2.337

**Published:** 2024-01-22

**Authors:** Koji Takahashi, Hiroshi Ohyama, Yuichi Takiguchi, Motoyasu Kan, Mayu Ouchi, Hiroki Nagashima, Kohichiroh Okitsu, Izumi Ohno, Naoya Kato

**Affiliations:** ^1^ Department of Gastroenterology Graduate School of Medicine Chiba University Chiba Japan; ^2^ Department of Medical Oncology Graduate School of Medicine Chiba University Chiba Japan

**Keywords:** ampullary carcinoma, duodenum, endoscopic retrograde cholangiopancreatography, penetration, straight biliary plastic stent

## Abstract

A 70‐year‐old woman presented to our hospital with abdominal discomfort. Gastrointestinal endoscopy revealed an ampullary tumor, while a biopsy revealed a pathological diagnosis of adenocarcinoma. No distant metastases were observed and neoadjuvant chemotherapy and surgical resection were planned. Shortly thereafter, she developed obstructive jaundice due to the ampullary carcinoma. The patient underwent endoscopic retrograde cholangiopancreatography, during which a straight plastic stent was placed in the bile duct. The patient was discharged without complications. Neoadjuvant chemotherapy was initiated. Two months later, she was readmitted for surgery while asymptomatic. Endoscopic retrograde cholangiopancreatography was scheduled to replace the stent with a nasobiliary drainage tube for the surgery. Endoscopic imaging revealed that the proximal end of the stent had penetrated the duodenum on the oral side of the ampullary carcinoma. The distal end of the stent was grasped with forceps and the stent was successfully removed. A catheter was inserted into the bile duct orifice and cholangiography was performed, which revealed that the distal bile duct and the duodenum had formed a fistula. A guidewire was placed in the bile duct via the papilla and a nasobiliary drainage tube was placed. After endoscopic retrograde cholangiopancreatography, the patient exhibited smooth progress without issue. Pancreaticoduodenectomy was performed on the fourth day after the nasobiliary drainage tube placement, and the patient's postoperative course was uneventful. The proximal end of a biliary stent penetrating the duodenal wall is an infrequent phenomenon. This case report highlights a rare but noteworthy adverse event associated with straight biliary plastic stent placement.

## INTRODUCTION

Straight‐type plastic biliary stents are widely used for the endoscopic treatment of biliary diseases. Although plastic stent placement in the bile duct is an effective and safe drainage procedure for obstructive jaundice and acute cholangitis, stent‐related adverse events have occasionally been reported. Well‐known adverse events associated with biliary plastic stent use include cholangitis, cholecystitis, and migration.^1^, ^2^ Although less common, stent‐induced contralateral duodenal wall perforation has also been reported.^3^, ^4^ This can be primarily attributed to injury of the duodenal wall by the distal end of a straight stent. Few reports of duodenal wall penetration by the proximal end of a straight stent have been published.^5^ Here we report a case in which the duodenal wall was penetrated by the proximal end of a straight plastic stent placed for obstructive jaundice caused by ampullary carcinoma.

## CASE REPORT

**FIGURE 1:**
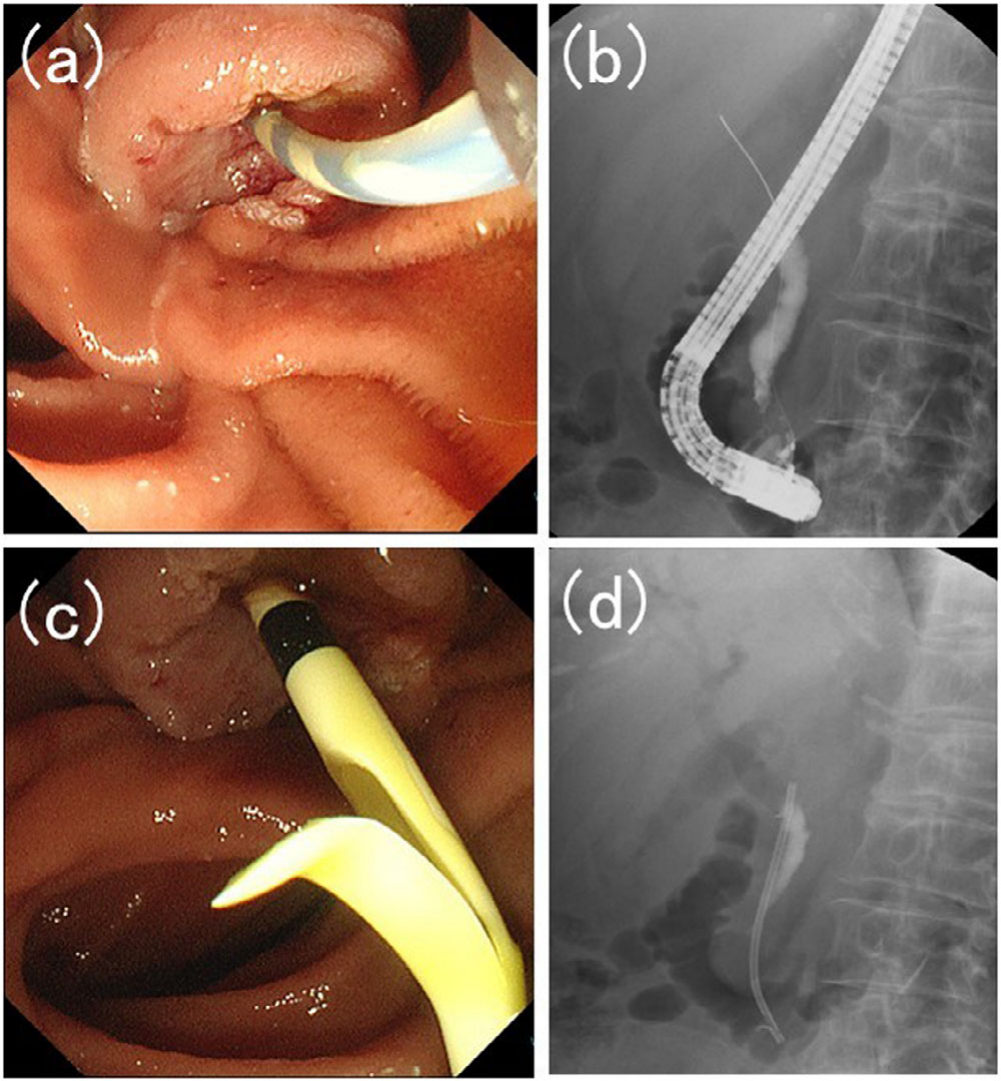
(a) The bile duct ostium was identified, and a catheter was inserted into the bile duct. (b) Cholangiography was performed, and a guidewire was placed in the bile duct. (c, d) A straight plastic stent was placed in the bile duct.

**FIGURE 2:**
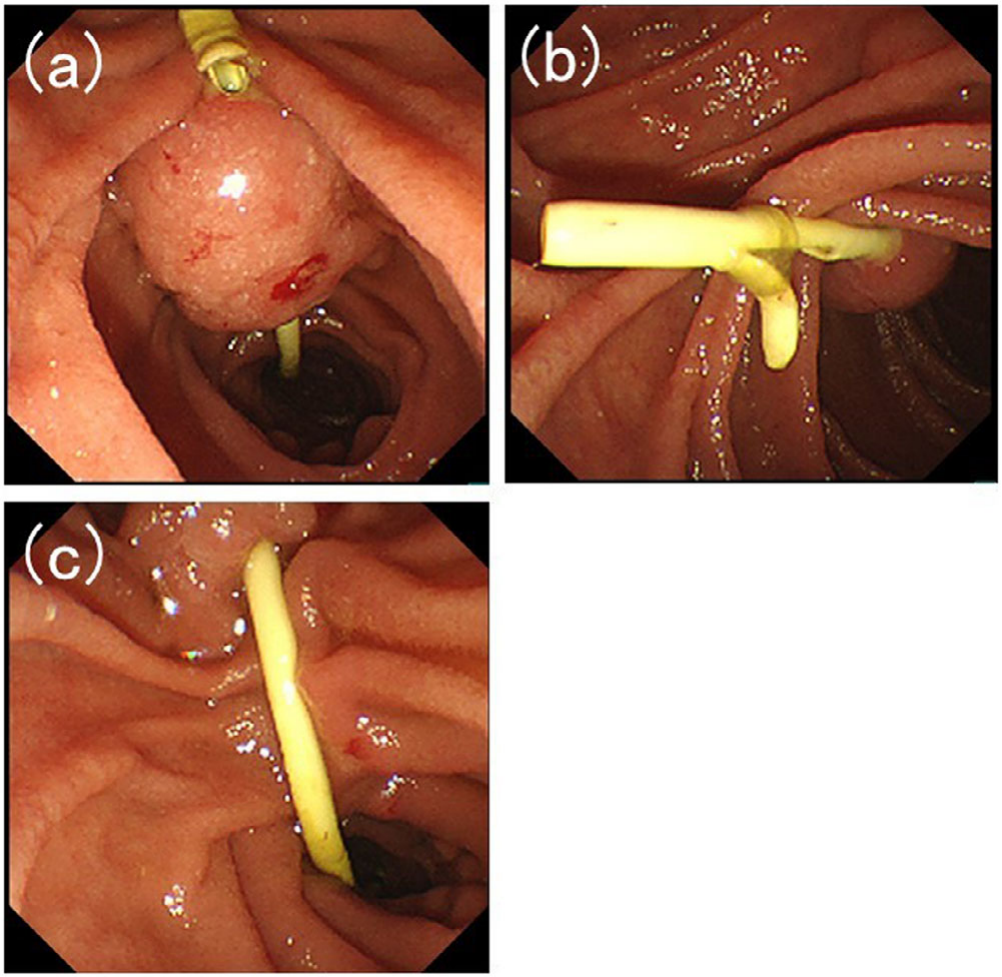
(a–c) When the endoscope was inserted, the proximal end of the stent penetrated the duodenal side on the oral side of the ampullary carcinoma.

**FIGURE 3:**
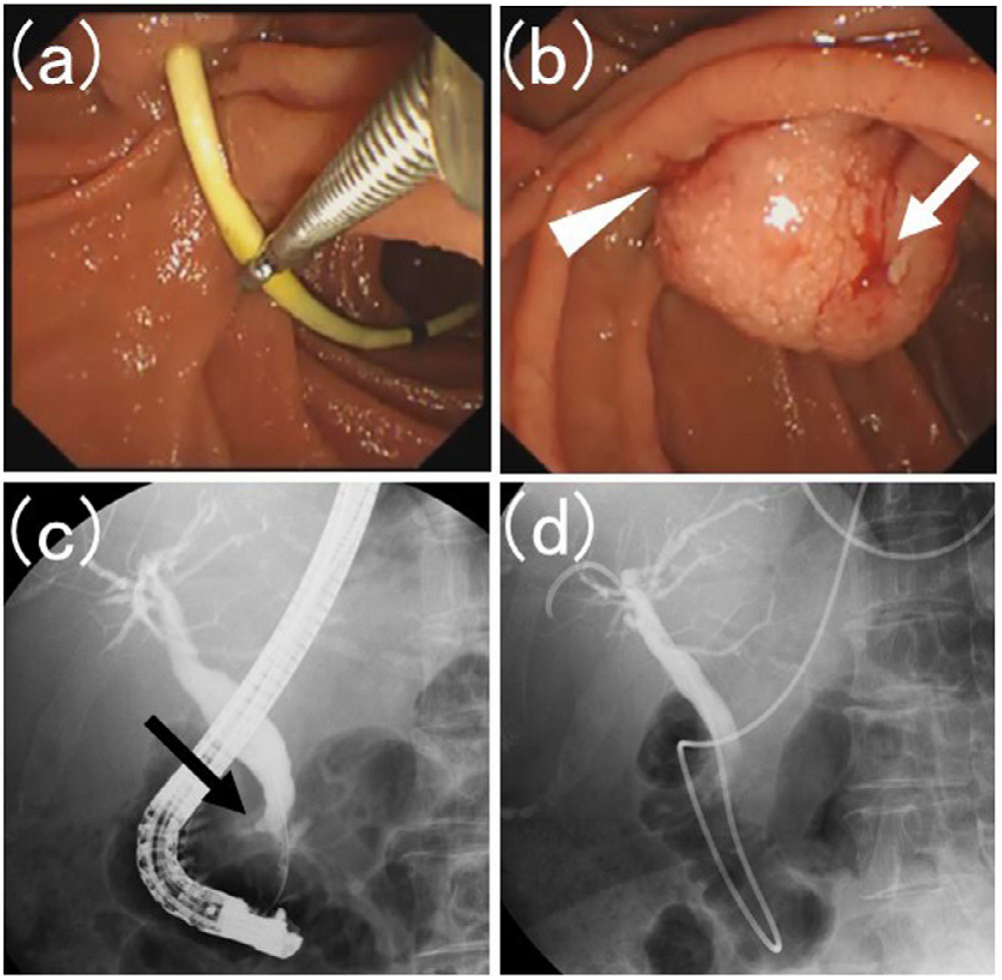
(a) The distal end of the stent was grasped with forceps and the stent pulled out successfully. (b) Almost no bleeding from the bile duct orifice (white arrow) was recorded. The white arrowhead indicates the point where the stent penetrated the duodenal wall. (c) A catheter was inserted into the bile duct orifice, and cholangiography was performed. The distal bile duct and duodenum formed a fistula (black arrow). (d) A nasobiliary drainage tube was placed.

A 70‐year‐old woman with abdominal discomfort visited a hospital near her home. Blood tests revealed elevated levels of hepatobiliary enzymes; however, jaundice was not diagnosed. Abdominal ultrasonography reveals bile duct dilation. She was referred to our hospital for further examination. Computed tomography revealed a mass in the ampulla of Vater. Gastrointestinal endoscopy revealed an ampullary tumor and a biopsy revealed a pathological diagnosis of adenocarcinoma. Endoscopic ultrasonography revealed that the tumor had spread beyond the muscular layer of the duodenum. No distant metastases were observed, and neoadjuvant chemotherapy and surgical resection were planned. Shortly thereafter, she developed obstructive jaundice due to ampullary carcinoma. Therefore, we decided to place a biliary stent via endoscopic retrograde cholangiopancreatography (ERCP). During ERCP, a straight plastic stent was placed in the bile duct (Figure 1). No endoscopic sphincterotomy was performed. The patient was discharged without complications. Neoadjuvant chemotherapy was initiated. Two months later, the patient was readmitted for surgery. She was asymptomatic. ERCP was scheduled to replace the stent with a nasobiliary drainage tube for surgery. Endoscopic imaging revealed that the proximal end of the stent had penetrated the duodenum on the oral side of the ampullary carcinoma (Figure 2). The distal end of the stent was grasped with forceps and the stent was successfully removed. Almost no bleeding was detected in the bile duct orifice or at the point where the stent had penetrated the duodenal wall. Subsequently, a catheter was inserted into the bile duct orifice, and cholangiography was performed. The distal bile duct and the duodenum form a fistula. A guidewire was placed in the bile duct via the papilla and an nasobiliary drainage tube was placed (Figure 3 and Video S1). After the ERCP, the disease progressed without complications. Pancreaticoduodenectomy was performed on the fourth day after the nasobiliary drainage tube placement, and the patient's postoperative course was uneventful. Pathological evaluation of the resected specimen revealed an adenocarcinoma. The adenocarcinoma remained within the mucosa and no lymph node metastasis or vascular invasion was noted. No problems related to stent penetration were observed.

## DISCUSSION

In this case, a straight plastic stent was transpapillarily placed in the bile duct for obstructive jaundice due to ampullary carcinoma. Then the proximal end of the stent penetrated the duodenal wall. The patient developed no abdominal or jaundice relapse. Some reports have described perforation of the contralateral duodenal wall at the distal end of a straight stent. However, reports of the proximal end of a biliary stent penetrating the duodenal wall, as in this case, are rare.

Transpapillary biliary stenting using ERCP was first reported by Sohendra in 1980.^4^ A plastic stent was used in this report. Such plastic stents are still widely used today. Plastic stents are manufactured in various shapes, and straight‐ and double‐pigtail stents are frequently used in routine clinical practice. To prevent migration, straight stents are usually provided with side flaps, whereas double‐pigtail stents are coiled at their proximal and distal ends.^7^, ^8^ A retrospective study in 2022 revealed that in cases in which the stent was retained for an extended duration, distal stent migration was more commonly observed with double‐pigtail versus straight stents.^9^

Among the adverse events associated with biliary stenting, stent‐induced duodenal wall injuries are rare. A retrospective study conducted in 2020 investigated 25,224 ERCP procedures and identified duodenal perforations in 11 patients (0.04%). Among them, plastic stents were used in 10 (91%), whereas metal stents were used in one (9%). Notably, all perforations were associated with stents positioned in the bile duct, while none were associated with stents placed in the pancreatic duct.^3^

**FIGURE 4:**
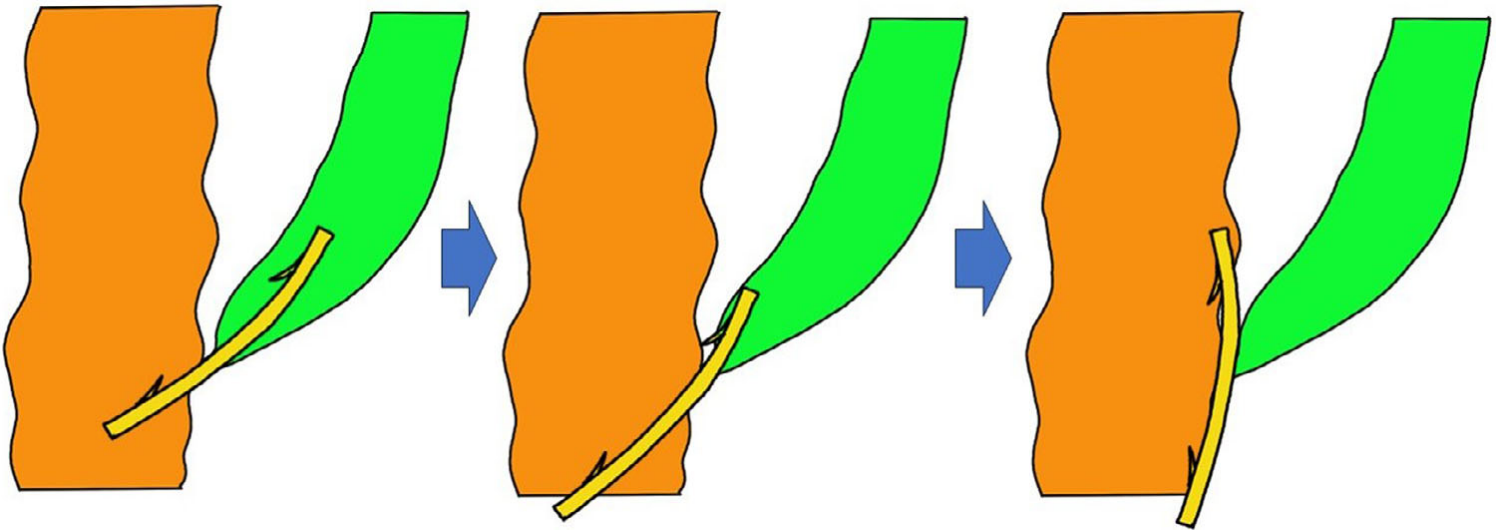
The mechanism of stent penetration in this case is demonstrated. First, the proximal flap of the placed stent was moved distally until it was just above the duodenal papilla. The proximal end of the stent then hit the bile duct wall just above the papilla. Finally, peristalsis of the duodenal wall pushed the distal end of the stent against the contralateral duodenal wall, penetrating the duodenal wall.

Figure 4 shows the inferred mechanism of stent penetration. First, the proximal flap of the stent was moved distally to just above the duodenal papilla. The proximal end of the stent hit the bile duct wall immediately above the papilla. Subsequently, the peristalsis of the duodenal wall pushed the distal end of the stent. Finally, the proximal end of the stent hit the bile duct wall; as a result, the stent penetrated the duodenal wall.

In this case, the bile duct just upstream of the duodenal papilla was dilated due to ampullary carcinoma. We believe that one reason for this phenomenon is the large space in the bile duct just upstream of the duodenal papilla, which allowed greater mobility at the proximal end of the stent. However, no reports showing that this phenomenon occurs frequently in ampullary carcinoma have been published, and this phenomenon occurs due to a combination of various factors. Factors include bile duct diameter and shape and indwelling stent diameter, length, and shape. In this case, neoadjuvant chemotherapy was administered; however, chemotherapy is commonly administered when a straight plastic stent is in place for biliary tract cancer, including ampullary carcinoma. Bile duct perforation is likely to occur when heavy particle therapy is performed with a metal bile duct stent in place. However, in this case, neither heavy particle therapy nor a metal stent was used. The effect of neoadjuvant chemotherapy on penetration remains unclear. In the present case, the same phenomenon could have been avoided with the use of a double‐pigtail stent. However, it is difficult to predict whether this phenomenon occurs prior to ERCP. We hypothesized that this phenomenon could be avoided with the use of a double‐pigtail stent. When placing a double‐pigtail stent, there is no specific recommendation regarding whether the proximal end should be placed in the extra‐ or intrahepatic bile duct per endoscopist preference. Furthermore, the proximal end of the stent was located at the bend of the extrahepatic bile duct on fluoroscopic imaging after stent placement. Even with a straight stent, it is possible that the placement of a longer stent than the one we placed would have been more stable, and no displacement would have occurred. However, it remains possible that the same phenomenon will occur; therefore, it is difficult to conclude that this method is effective. The patient had no symptoms suggestive of intra‐ or retroperitoneal bile leakage, and her course was asymptomatic. We believe that the fistula formed slowly with stimulation by the proximal end of the stent, and it is unlikely that bile spread into the peritoneal cavity or retroperitoneum. Although cancer seeding was possible, considering that preoperative chemotherapy was administered and the fistula formation area was within the resection area, we believe that the possibility of cancer seeding was extremely low.

In conclusion, here we reported a case of duodenal wall penetration by the proximal end of a straight plastic stent placed in a case of obstructive jaundice secondary to ampullary carcinoma. This adverse event with straight biliary plastic stent use is rare but noteworthy.

## CONFLICT OF INTEREST STATEMENT

None.

## Supporting information

Video S1 **Endoscopic removal of a stent whose proximal end penetrated the duodenal wall**. Endoscopic imaging revealed that the proximal end of the stent had penetrated the duodenum on the oral side of the ampullary carcinoma. The distal end of the stent was grasped with forceps and the stent was removed. Thus, the stent was successfully removed. Almost no bleeding was detected in the bile duct orifice or at the point where the stent had penetrated the duodenal wall. A catheter was then inserted into the bile duct orifice, and a nasobiliary drainage tube was placed.
